# Preferences for training on mandatory reporting of intimate partner violence among healthcare professionals: a Norwegian cross-sectional study

**DOI:** 10.3389/fpubh.2026.1884227

**Published:** 2026-07-15

**Authors:** Veronica Kvalen Pilskog, John C. Besley, Thea Beate Brevik

**Affiliations:** 1 Department of Communication, Volda University College, Volda, Norway; 2 Department of Media and Communication, University of Oslo, Oslo, Norway; 3 College of Communication Arts and Sciences, Michigan State University, East Lansing, MI, United States; 4 Faculty of Health Sciences and Social Care, Molde University College, Molde, Norway

**Keywords:** domestic violence, duty to report, intimate partner violence, mandatory reporting, training

## Abstract

**Background:**

Training healthcare professionals to recognize and respond to intimate partner violence (IPV) is an important component of health system responses to violence. However, little research has explored how healthcare professionals prefer such training to be delivered.

**Objectives:**

This study explored healthcare professionals’ preferences for training modalities on mandatory reporting of IPV and the associations between participant characteristics and preferred training modalities.

**Materials and methods:**

A cross-sectional survey was conducted between May and September 2025 (*N* = 266) among healthcare professionals working in Norway. The questionnaire assessed demographic and professional characteristics, previous training on mandatory reporting of IPV, perceived competence, and preferences for training delivery formats. Descriptive statistics were used to summarize participant characteristics and teaching preferences. Logistic regression analyses explore associations between participant characteristics and preferred training modalities.

**Results:**

In-person meetings, courses, and workshops were the most frequently preferred training formats (62%), followed by e-learning (54%) and digital meetings and webinars (44%). Greater perceived time constraints were associated with a stronger preference for digital training formats. Less experienced professionals were more likely to prefer video-based and e-learning formats. Physicians and healthcare workers were less likely to prefer in-person training.

**Conclusion:**

Healthcare professionals’ preferences for training modalities on mandatory reporting of IPV vary across professional and contextual factors. Flexible and multimodal training strategies may increase participation and engagement in such training.

## Introduction

Intimate partner violence (IPV) is a serious global public health problem (1, 2). IPV includes physical and sexual violence, stalking, and psychological aggression by a current or former intimate partner (3). Globally, an estimated 27% of women aged 15–49 who have been in a relationship report having experienced physical and/or sexual IPV in their lifetime (4). The WHO (2) estimates that one in three women will experience such violence at some point in their lifetime. Although women are disproportionately affected by IPV and are more likely to experience severe forms of violence, men may also experience IPV. Norwegian data indicate that approximately 16% of women and 15% of men report having experienced physical violence from an intimate partner, although severe partner violence is substantially more prevalent among women (5). IPV is associated with a wide range of adverse health outcomes, including physical injury, chronic disease, and mental illness (6), and, in its most extreme form, intimate partner homicide (7).

Several countries have implemented legislation requiring specific professionals or members of the public to report ongoing or future IPV (1), however, mandatory reporting legislation varies considerably across countries and jurisdictions regarding which forms of violence should be reported, how reporting should occur, and who is obliged to report suspected cases (1, 8). In Norway, mandatory reporting laws apply to all individuals who have reason to believe it is *certain or most likely* that the IPV is occurring or might occur (9). This places healthcare professionals in an important position, as they are often among the first to identify signs of IPV.

Despite their central role, research indicates that many healthcare professionals lack knowledge about the reporting legislation and have limited experience applying these legal requirements (7). Insufficient knowledge and training may make it difficult for professionals to assess suspected cases and respond appropriately (10). This underscores the importance of equipping healthcare professionals with IPV-specific knowledge, practical skills, and legal literacy (11). In Norway, this responsibility is further reinforced by the Health Personnel Act (1999, §4), which requires healthcare professionals to maintain up-to-date medical knowledge and competencies in order to provide safe and effective care.

Training and continuing education have emerged as key strategies to improve healthcare professionals’ ability to identify and respond to IPV. The WHO (2) highlights the training of healthcare professionals as a critical component of responses to sexual and gender-based violence, as it supports survivor-centered care, minimizes the risk of further harm, and strengthens the overall capacity of health systems in crisis situations. Evidence from intervention studies suggests that IPV training may improve knowledge, attitudes, and perceived readiness to respond (12). Similarly, Ross et al. (13) found that an education program for registered nurses increased knowledge and confidence in sexual violence screening and care. Targeted training has also been demonstrated to improve knowledge of legal frameworks and reporting criteria in a Norwegian context (14).

In addition to knowledge and attitudes, healthcare professionals’ self-efficacy in responding to IPV is central to effective practice. Self-efficacy refers to individuals’ beliefs in their capability to organize and execute the actions required to manage prospective situations (15). A systematic review suggests that self-efficacy may act as a mechanism linking theoretical knowledge to clinical practice, helping healthcare professionals translate acquired knowledge into action (27). Research demonstrates that healthcare professionals’ engagement with knowledge and clinical practice is often constrained by factors such as time limitations, information overload, limited organizational support, and self-efficacy (16). These constraints may also influence how professionals engage with and apply IPV-related training in practice.

However, IPV training varies substantially in terms of content, duration, and delivery format. Research suggests that interactive and practice-oriented approaches may be associated with improved learning outcomes compared with purely didactic methods (17). At the same time, structural barriers such as limited time, institutional support, and available resources may constrain participation in training (18). A literature review by Kirk and Bezzant (19) similarly identified lack of training, time, privacy, guidelines, policies, and employer support as barriers preventing health professionals from screening women for domestic abuse. Digital formats, including webinars and e-learning, have been proposed as more flexible alternatives and may improve knowledge and self-efficacy, although evidence remains mixed and heterogeneous (28, 29). A recent cross-sectional study found that healthcare professionals reported lower levels of self-efficacy in intervention and referral than in other aspects of IPV management, highlighting the need for targeted training strategies in these areas (20). Overall, the existing literature indicates substantial variation in both the design and effectiveness of IPV training interventions across professional groups and contexts (21).

Although existing research has demonstrated the importance of training, to our knowledge, no studies have examined how healthcare professionals prefer such training to be delivered. Understanding these preferences may increase the likelihood that professionals participate in training, engage with the training content, and apply what they learn in their daily practice. This is particularly relevant in the context of IPV, where both legal knowledge and practical competence are required to ensure appropriate responses to suspected cases.

The aim of this study was to explore healthcare professionals’ preferences for training modalities related to mandatory reporting of IPV.

The study addressed the following research questions:

(1) Which training delivery formats do healthcare professionals preferfor training on mandatory reporting of IPV?(2) Which demographic and professional characteristics are associated with these preferences?

## Materials and methods

This study employed a cross-sectional survey to explore healthcare professionals’ preferences for training modalities and how these preferences varied across demographic and professional characteristics. The study formed part of a larger interdisciplinary research project (Project Number 313902) funded by the Norwegian Research Council and approved by the Norwegian Agency for Shared Services in Education and Research (details blinded for peer review).

### Recruitment procedure

Eligible participants were healthcare professionals currently working in Norway, regardless of profession, workplace setting, or years of experience. Participation was voluntary and anonymous.

Data were collected between May and September 2025 using Nettskjema, a secure Norwegian web-based survey platform. Participants were recruited primarily from one Norwegian region through continuing education programs, hospital-based specialist healthcare services, and professional networks. The survey was also disseminated through the authors’ professional networks, including social media and direct messaging, which may have reached healthcare professionals in other parts of Norway.

### Survey

The participants were invited to participate in a new training program for healthcare professionals, where they would learn how to recognize and respond to signs of violence, as well as how to act in situations where there may be a legal obligation to report, in accordance with Norwegian legislation.

The questionnaire assessed: (1) demographic and professional characteristics (gender, age, profession, primary workplace, and years of experience); (2) training experience and availability for training in mandatory reporting of IPV; (3) self-efficacy includingskills for participating in training and skills in mandatory reporting of IPV. The former reflect respondents’ perceived readiness to engage in training, while the latter refers to their perceived skills in understanding and applying legal reporting obligations; and (4) preferences for training delivery formats (e.g., webinars, e-learning modules, videos, infographics, written materials, and in-person workshops). Participants could select multiple options when indicating preferred formats.

The median survey completion time, based on completion times automatically recorded by the survey platform, was approximately 4 min. The questionnaire was developed in English and translated into Norwegian by the research team. During the translation process, selected English terms and phrases were discussed with an Associate Professor in Norwegian didactics to enhance linguistic clarity and ensure conceptual equivalence. Prior to data collection, the questionnaire was pilot tested with a small group to assess clarity, relevance, and usability. Based on their feedback, minor revisions were made to the wording and structure of selected items before the final version was distributed.

### Statistical analysis

Descriptive statistics were used to summarize participant characteristics and preferences for different teaching modalities. Associations between participant characteristics and preferred teaching modalities (digital meetings/webinar, e-learning/online courses, videos, and in-person meetings/courses/workshops) were analyzed using logistic regression. As participants could select multiple modalities, the outcomes were not mutually exclusive and each modality was therefore coded as a separate binary outcome (preferred/not preferred) and analyzed independently. Given the multiple analyses conducted, these were considered exploratory in nature. The analytical strategy followed the purposeful selection approach described by Hosmer Jr et al. (22).

For each outcome, univariable logistic regression analyses were conducted for all participant characteristics. Results are reported as odds ratios (ORs) and 95% confidence intervals (CIs). Covariates with *p* ≤ 0.20 were subsequently entered into multivariable logistic regression models to estimate adjusted associations. This liberal screening threshold was applied to avoid the premature exclusion of potentially important explanatory or confounding variables (22). Self-efficacy variables were included in the univariable analyses but did not meet the inclusion criterion (*p* ≤ 0.20) for entry into the multivariable models and were therefore not retained in subsequent analyses.

The full models were then reduced to obtain more parsimonious and stable final models. Backward elimination was applied, retaining covariates with *p* ≤ 0.05 or those whose removal resulted in a ≥ 20% change in regression coefficients, suggesting potential confounding (22). This iterative process of variable removal and model refitting continued until all retained variables were considered clinically and/or statistically relevant.

Likelihood ratio (LR) tests were used to compare the full and final models. As no significant loss of fit was observed for any of the four outcomes, the final models were considered to provide an adequate representation of the data. In addition, multicollinearity was assessed using variance inflation factors (VIFs) in the full multivariable models for each outcome. Mean VIF values across outcomes ranged from 1.24 to 1.55, with individual VIFs ranging from 1.06 to 2.69. As no covariate exceeded a VIF of 5, multicollinearity was considered acceptable.

### Ethical considerations

All participants were presented with written information about the study on the first page of the online survey. The information described the study’s purpose, the voluntary nature of participation, and the type of data collected. Participants were required to provide electronic informed consent before proceeding to the survey questionnaire.

Survey data were stored on secure servers and subsequently transferred to institutionally approved research servers. Only the authors had access to the dataset. No identifying information was collected. The study was registered with the Norwegian data protection authority Sikt (reference number 833497).

## Results

The results are presented in three sections: (1) sample characteristics, (2) preferred training modalities, and (3) associations between participant characteristics and training preferences.

### Sample characteristics

A total of 266 healthcare professionals completed the survey, and there were no missing responses. Descriptive characteristics of the sample are presented in Table 1. Most participants were women (86%, *n* = 229). The most common age groups were 25–34 years (40%, *n* = 106) and 35–44 years (33%, *n* = 88). The largest professional groups were nurses (44%, *n* = 117), physicians (17%, *n* = 45), and psychologists (8%, *n* = 21). Over half of the participants worked in hospitals (51%, *n* = 136), followed by municipal health services (32%, *n* = 85). Most respondents reported no prior training on mandatory reporting of IPV (61%, *n* = 162). Self-efficay related to participating in training was generally rated as high (49%, *n* = 130) or very high (26%, *n* = 68). In contrast, self-efficacy related to mandatory reporting of IPV was lower, with most participants reporting low (36%, *n* = 95) or moderate (46%, *n* = 122) levels.

**Table 1:** Sample characteristics (*n* = 266).

Variable	Category	*n*	%
Time	Not at all	16	6.02
	To a small extent	66	24.81
	Neither nor / neutral	83	31.20
	To a large extent	87	32.71
	To a very large extent	14	5.26
Skills	Not at all	0	0
	To a small extent	11	4.14
	Neither nor / neutral	57	21.43
	To a large extent	130	48.87
	To a very large extent	68	25.56
Skills (MR-IPV)	Very low	15	5.64
	Low	95	35.71
	Moderate	122	45.86
	High	30	11.28
	Very high	4	1.50
Channel	Digital meetings or webinars	118	44.36
	E-learning or online courses	144	54.14
	Email	17	6.39
	Short videos	101	38.11
	Infographics or posters	14	5.26
	In-person meetings, courses, or workshops	166	62.41
Profession	Physician	45	16.92
	Nurse	118	44.36
	Psychologist	22	8.27
	Social educator	15	5.64
	Physiotherapist	8	3.01
	Mid-wife	16	6.02
	Healthcare worker	6	2.26
	Other	36	13.53
Work experience	< 1 year	19	7.14
	1–3 years	49	18.42
	4–7 years	53	19.92
	6–15 years	88	33.08
	> 15 years	57	21.43
Primary workplace	Hospital	136	51.13
	Municipal health services	86	32.33
	Specialist health services	22	8.27
	Private practice	7	2.63
	Other	15	5.64
Prev. training	0 days	162	60.90
	Less than 1 day	54	20.30
	1–2 days	33	12.41
	3–5 days	11	4.14
	> 5 days	6	2.26
Gender	Women	229	86.09
	Men	37	13.91
Age group	< 25 years	17	6.39
	25–34 years	106	39.85
	35–44 years	88	33.08
	45–54 years	34	12.78
	55–64 years	14	5.26
	≥ 65 years	7	2.63

### Preferred training formats on mandatory reporting of intimate partner violence

**Figure 1:**
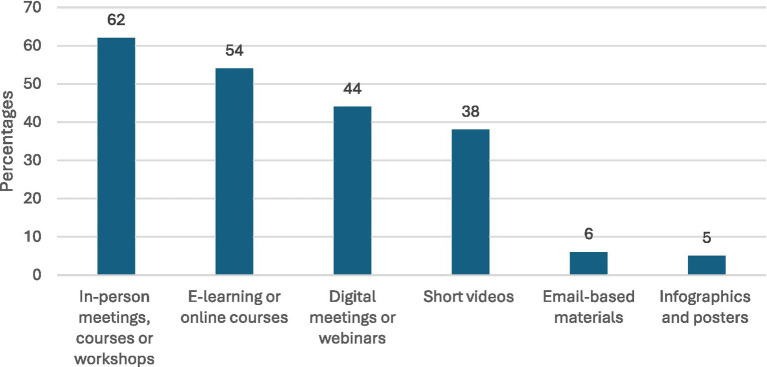
Preferred formats for training on mandatory reporting of intimate partner violence.

Participants were asked to indicate their preferred formats for training on mandatory reporting of IPV (Figure 1). In-person meetings, courses, and workshops were the most frequently preferred training modality, selected by 62% (*n* = 166) of respondents. E-learning or online courses were preferred by 54% (*n* = 144), while 44% (*n* = 118) indicated a preference for digital meetings or webinars. Short instructional videos were preferred by 38% (*n* = 101) of respondents. By contrast, fewer participants preferred receiving information through email-based materials (6%, *n* = 17) or infographics and posters (5%, *n* = 14).

### Variation in training preferences across participant characteristics

Logistic regression analyses were conducted to explore associations between participant characteristics and preferences for different training formats (Tables 2, 3). As noted, each format was analyzed as a separate outcome variable. The limited preference for email-based materials and infographics/posters made these formats unsuitable for regression analyses; they were therefore not included in the analyses below.

**Table 2:** Univariable analyses of the of four outcomes.

Variable	Digital meetings or webinars		E-learning or online courses		Short videos		In-person meetings, courses or workshops	
	OR (95% CI)	*p*	OR (95% CI)	*p*	OR (95% CI)	*p*	OR (95% CI)	*p*
Time	1.32 (1.04–1.69)	**0.025**	1.50 (1.17–1.93)	**0.001**	1.20 (0.94–1.54)	**0.152**	1.98 (0.76–1.25)	0.840
General knowledge	1.10 (0.81–1.49)	0.548	1.09 (0.81–1.49)	0.541	0.92 (0.78–1.26)	0.612	1.22 (0.89–1.65)	0.211
Knowledge today	0.99 (0.73–1.34)	0.950	0.89 (0.66–1.20)	0.454	0.89 (0.75–1.21)	0.448	0.98 (0.72–1.34)	0.913
Profession (ref: nurse)		0.766		**0.072**		0.459		**0.004**
Physician	0.77 (0.38–1.56)	0.468	1.18 (0.59–2.38)	0.639	1.10 (0.54–2.24)	0.797	0.33 (0.16–0.67)	0.**002**
Psychologist	0.88 (0.35–2.21)	0.784	0.45 (0.18–1.15)	**0.197**	1.00 (0.39–2.57)	0.994	1.68 (0.58–4.89)	0.342
Social educator	2.54 (0.82–7.88)	**0.107**	0.53 (0.18–1.57)	0.249	1.99 (0.68–5.88)	0.212	1.97 (0.53–7.41)	0.312
Physiotherapist	1.27 (0.30–5.32)	0.744	5.51 (0.66–46.25)	**0.116**	2.91 (0.66–12.77)	**0.158**	0.49 (0.12–2.08)	0.336
Midwife	0.99 (0.34–2.83)	0.981	0.47 (0.16–1.39)	**0.172**	0.40 (0.11–1.49)	**0.173**	1.09 (0.35–3.34)	0.886
Healthcare worker	1.27 (0.25–6.55)	0.776	3.94 (0.45–34.76)	0.217	1.74 (0.34–9.02)	0.507	0.10 (0.01–0.87)	**0.037**
Other	1.01 (0.48–2.15)	0.968	0.88 (0.42–1.86)	0.739	1.11 (0.52–2.40)	0.790	0.99 (0.45–2.18)	0.975
Work experience (ref: more than 15 years)		0.553		0.282		**0.031**		0.686
Less than one year	0.60 (0.21–1.76)	0.355	1.07 (0.38–3.04)	0.894	0.67 (0.19–2.32)	0.524	2.04 (0.65–6.42)	0.225
1–3 years	0.60 (0.28–1.31)	**0.201**	2.24 (1.02–4.92)	**0.044**	3.07 (1.37–6.88)	0.**007**	1.50 (0.68–2.32)	0.318
4–7 years	0.74 (0.35–1.56)	0.423	1.68 (0.79–3.57)	**0.178**	1.64 (0.74–3.65)	0.225	1.20 (0.56–2.58)	0.640
6–15 years	0.99 (0.51–1.93)	0.976	1.31 (0.67–2.55)	0.434	1.50 (0.73–3.09)	0.272	1.10 (0.56–2.17)	0.780
Workplace (ref: nurse)		0.886		**0.170**		0.233		0.227
Municipal health services	0.87 (0.51–1.51)	0.630	1.50 (0.86–2.60)	**0.150**	1.51 (0.87–2.64)	**0.147**	1.19 (0.68–2.09)	0.540
Specialist health services	0.80 (0.32–2.00)	0.637	0.62 (0.25–1.54)	0.298	0.93 (0.36–2.45)	0.889	1.37 (0.52–3.58)	0.522
Private practice	0.46 (0.08–2.47)	0.368	0.36 (0.07–1.90)	**0.226**	1.50 (0.32–6.99)	0.606	3.82 (0.45–32.72)	0.220
Other	1.01 (0.35–2.95)	0.980	0.78 (0.27–2.26)	0.645	3.00 (1.01–8.95)	**0.049**	0.43 (0.14–1.26)	**0.124**
Previous training (ref: none)		0.374		0.655		0.819		0.524
Less than one day	1.25 (0.67–2.32)	0.477	1.32 (0.70–2.48)	0.385	1.10 (0.59–2.06)	0.771	0.57 (0.30–1.06)	**0.077**
1–2 days	0.77 (0.35–1.67)	0.509	0.70 (0.33–1.49)	0.354	0.91 (0.42–1.98)	817	0.92 (0.42–2.02)	0.844
3–5 days	2.36 (0.66–8.38)	0.**184**	0.70 (0.21–2.39)	0.570	0.91 (0.26–3.25)	0.887	0.92 (0.26–3.29)	0.904
More than 5 days	2.70 (0.48–15.14)	0.260	0.84 (0.16–4.29)	0.835	0.32 (0.04–2.80)	0.302	1.06 (1.19–5.95)	0.950
Gender (ref: women)	0.48 (0.23–1.02)	**0.057**	0.60 (0.30–1.21)	**0.155**	0.86 (0.42–1.78)	0.688	1.75 (0.81–3.79)	**0.156**
Age	1.04 (0.84–1.30)	0.709	0.86 (0.68–1.07)	**0.171**	0.82 (0.65–1.04)	**0.100**	0.87 (0.69–1.09)	0.227

Bold values indicate statistically significant associations (*p* < 0.05).

**Table 3:** Multivariable models of the four outcomes.

Variable	Full multivariable model		Final multivariable model	
	OR (95% CI)	*p*	OR (95% CI)	*p*
Digital meetings or webinars				
Time	1.41 (1.07–1.85)	0.013	1.32 (1.03–1.69)	**0.030**
Profession (ref: nurse)				
Physician	1.00 (0.47–2.13)	0.994		
Psychologist	0.87 (0.33–2.30)	0.779		
Social educator	3.15 (0.92–10.78)	0.068		
Physiotherapist	0.84 (0.19–3.81)	0.823		
Midwife	0.76 (0.23–2.46)	0.642		
Healthcare worker	1.09 (1.20–6.08)	0.922		
Other	0.85 (0.39–1.86)	0.682		
Work Experience (ref: more than 15 years)				
Less than one year	0.64 (0.21–1.93)	0.428		
1–3 years	0.49 (0.21–1.14)	0.197		
4–7 years	0.69 (0.21–1.56)	0.371		
6–15 years	1.11 (0.55–2.24)	0.775		
Previous training (ref: non)				
Less than one day	1.12 (0.58–2.17)	0.730		
1–2 days	0.62 (0.27–1.41)	0.253		
3–5 days	2.01 (0.53–7.62)	0.304		
More than 5 days	237 (0.35–16.17)	0.377		
Gender (ref: women)	0.49 (0.22–1.08)	0.077	0.49 (0.23–1.05)	0.066
Likelihood ratio test: LR χ^2^(15) = 12.69; *p* = 0.626				
E-learning or online courses				
Time	1.60 (1.20–2.13)	0.001	1.52 (1.17–1.96)	**0.002**
Profession (ref: nurse)				
Physician	1.61 (0.74–3.48)	0.226		
Psychologist	0.43 (0.15–1.24)	0.119		
Social educator	0.39 (0.12–1.30)	0.126		
Physiotherapist	3.77 (0.42–33.77)	0.236		
Midwife	0.48 (0.15–1.50)	0.205		
Healthcare worker	3.58 (0.37–34.41)	0.270		
Other	0.76 (0.32–1.80)	0.529		
Work experience (ref: more than 15 years)				
Less than one year	1.21 (0.31–4.76)	0.780	1.19 (0.41–3.44)	0.751
1–3 years	2.53 (0.84–7.61)	0.100	2.27 (1.02–5.06)	**0.045**
4–7 years	1.78 (0.64–4.97)	0.274	1.79 (0.83–3.87)	0.140
6–15 years	1.54 (0.65–3.69)	0.327	1.58 (0.79–3.17)	0.196
Workplace (ref: hospital)				
Municipal health services	1.26 (0.66–2.39)	0.483		
Specialist health services	0.66 (0.24–1.77)	0.405		
Private practice	0.46 (0.07–2.84)	0.402		
Other	0.88 (0.25–3.00)	0.835		
Gender (ref: women)	0.62 (0.29–1.34)	0.227		
Age	1.06 (0.75–1.50)	0.723		
Likelihood ratio test: LR χ^2^(15) = 19.79; *p* = 0.101				
Short videos				
Time	1.15 (0.87–1.52)	0.326	1.19 (0.92–1.54)	0.187
Profession (ref: nurse)				
Physician	1.28 (0.59–2.79)	0.528		
Psychologist	0.89 (0.31–2.58)	0.830		
Social educator	1.37 (0.43–4.36)	0.599		
Physiotherapist	2.50 (0.53–11.88)	0.259		
Midwife	0.47 (0.12–1.86)	0.284		
Healthcare worker	1.82 (0.33–9.92)	0.490		
Other	0.84 (0.35–2.01)	0.691		
Work experience (ref: more than 15 years)				
Less than one year	0.61 (0.13–2.77)	0.523	0.69 (0.20–2.42)	0.565
1–3 years	2.67 (0.90–7.99)	0.080	3.06 (1.36–6.89)	**0.007**
4–7 years	1.37 (0.48–3.95)	0.557	1.68 (0.75–3.74)	0.207
6–15 years	1.57 (0.63–3.89)	0.329	1.62 (0.78–3.38)	0.198
Workplace (ref: hospital)				
Municipal health services	1.28 (0.68–2.41)	0.438		
Specialist health services	1.00 (0.36–2.75)	0.996		
Private practice	1.75 (0.32–9.53)	0.515		
Other	4.10 (1.17–14.37)	0.028		
Age	0.89 (0.63–1.26)	0.503		
Likelihood ratio test: LR χ^2^(15) = 10.79; *p = 0*.547				
In-person meetings, courses or workshops				
Q10 Profession (ref: nurse)				
Physician	0.20 (0.13–0.63)	*0.*002	0.32 (0.15–0.65)	**0.002**
Psychologist	1.73 (0.53–5.60)	0.362	1.73 (0.56–5-36)	0.339
Social educator	2.45 (0.60–10.11)	0.214	2.44 (0.61–9.76)	0.206
Physiotherapist	0.57 (0.12–2.63)	0.468	0.048 (0.11–2.10)	0.328
Midwife	1.05 (0.31–3.53)	0.937	1.04 (0.33–3.26)	0.942
Healthcare worker	0.10 (0.01–0.97)	0.047	0.94 (0.01–0.84)	**0.035**
Other	1.35 (0.55–3.32)	0.513	1.21 (0.50–2.91)	0.671
Work experience (ref: more than 15 years)				
Less than one year	2.53 (0.74–8.63)	0.138		
1–3 years	1.36 (0.56–3.29)	0.501		
4–7 years	1.29 (0.54–3.08)	0.571		
6–15 years	1.08 (0.51–2.26)	0.843		
Workplace (ref: hospital)				
Municipal health services	1.08 (0.55–2.14)	0.827	0.99 (0.52–1.85)	0.964
Specialist health services	0.98 (0.34–2.80)	0.973	0.94 (0.34–2.59)	0.908
Private practice	2.52 (0.26–24.13)	0.424	2.72 (0.28–26.23)	0.386
Other	0.27 (0.07–0.98)	0.047	0.25 (0.07–0.83)	**0.024**
Previous training (ref: none)				
Less than one day	0.64 (0.32–1.21)	0.227		
1–2 days	1.16 (0.48–2.83)	0.743		
3–5 days	1.24 (0.30–5.11)	0.769		
More than 5 days	1.70 (0.22–13.28)	0.613		
Gender (ref: women)	1.91 (0.81–4.53)	0.142		
Likelihood ratio test: LR χ^2^(15) = 7.30; *p = 0*.606				

Bold values indicate statistically significant associations (*p* < 0.05).

#### Preferences for digital meetings or webinars

Perceived time constraints were associated with preferences for digital meetings or webinars. Respondents reporting greater time constraints had higher odds of preferring digital meetings compared to those reporting fewer time constraints (OR = 1.3, 95% CI: 1.0–1.7, *p* = 0.03). Gender was also associated with these preferences, with male respondents reporting lower odds of preferring digital meetings than female respondents, although this association did not reach statistical significance (OR = 0.5, 95% CI: 0.2–1.0, *p* = 0.07).

#### Preferences for e-learning or online courses

Preferences for e-learning or online courses were associated with both perceived time constraints and work experience. Respondents reporting greater time constraints had higher odds of preferring e-learning formats (OR = 1.5, 95% CI: 1.2–2.0, *p* < 0.01). Respondents with 1–3 years of professional experience were more likely to prefer e-learning than those with more than 15 years of experience (OR = 2.3, 95% CI: 1.0–5.1, *p* = 0.05).

#### Preferences for short videos

Preferences for short videos were associated with work experience. Respondents with 1–3 years of work experience had higher odds of preferring video-based training than those with more than 15 years of experience (OR = 3.1, 95% CI: 1.4–6.9, *p* < 0.01).

#### Preferences for in-person meetings, courses, or workshops

Preferences for in-person meetings, courses, or workshops were associated with both professional group affiliation and workplace context. Compared to nurses, physicians reported lower odds of preferring in-person meetings (OR = 0.3, 95% CI: 0.2–0.6, *p* < 0.01). Healthcare workers also reported significantly lower odds of preferring in-person meetings, (OR = 0.1, 95% CI: 0.0–0.8, *p* = 0.04).

Workplace context was also associated with training preferences, although this association was observed only among respondents who reported working in workplace settings *other* than municipal health services, specialist health services, or private practice (OR = 0.3, 95% CI: 0.1–0.8, *p* = 0.02). As the “other” category was not further specified, it was not possible to determine which workplace settings this group represented.

## Discussion

This study explored healthcare professionals’ preferences for training formats on mandatory reporting of intimate partner violence. Four findings emerged from the analysese. First, in-person meetings, courses, and workshops were the most frequently preferred training format. Second, perceived time constraints were associated with a greater preference for digital training formats (webinars and e-learning). Third, less experienced professionals were more likely to prefer video-based and online learning formats. Finally, physicians and healthcare workers were less likely to prefer in-person training.

Moreover, although self-efficacy were measured, it was not retained in the final multivariable models because it did not meet the predefined inclusion criteria in the purposeful selection procedure. However, self-efficacy remains conceptually relevant to the study, and the brevity of our survey may have limited our ability to capture this construct in sufficient detail. Therefore, the lack of association between training preferences and self-efficacy should be interpreted with caution and explored further in future studies.

### In-person formats most preferred

The preference for in-person training formats may be consistent with a perceived need for interactive and practice-oriented learning when dealing with complex and sensitive issues such as IPV. Responding to suspected violence involves not only knowledge of legal frameworks, but also communication skills, ethical judgment, and clinical decision-making under conditions of uncertainty. In this context, face-to-face formats may be perceived as better suited for discussion, reflection, and skills-based learning. This interpretation is consistent with research suggesting that interactive and practice-oriented approaches are associated with improved learning outcomes in IPV-related education (17). A recent scoping review further highlights that IPV training frequently incorporates experiential learning strategies such as role playing and scenario-based exercises to develop practical competencies (23). Although this evidence is derived primarily from student populations, it may be also transferable to clinical contexts in which complex communication and decision-making skills are required. The findings may suggest that opportunities for engagement and practical application are relevant considerations when selecting training formats related to IPV.

### Time constraints and digital formats

The association between perceived time constraints and preference for digital training formats may suggest that feasibility plays a central role in shaping training preferences. Rather than reflecting a clear preference for digital learning per se, this finding may indicate that flexible and accessible formats are necessary to accommodate competing clinical demands. In this context, webinars and e-learning may be perceived as more manageable due to their potential for asynchronous access and reduced time commitment. This is consistent with previous research identifying limited time as a key barrier to IPV-related training (18). At the same time, the findings may indicate that structural factors, such as workload and time availability, are associated with how different training formats are valued. Evidence on digital IPV education suggests that such approaches may improve knowledge and self-efficacy, although findings remain heterogeneous (28).

### Early-career professionals

Less experienced professionals demonstrated a greater preference for video-based and digital learning formats. This finding may be related to familiarity with different learning environments, including digital platforms. It is also possible that early-career professionals place greater value on flexible and self-paced learning formats when developing competence in complex areas such as IPV. Research on digital education suggests that online and video-based formats can support knowledge acquisition and self-efficacy (28). However, as existing research has not directly explored how training preferences vary by level of professional experience, this interpretation remains tentative. Nevertheless, the finding suggest that training preferences may vary across career stage.

### Physicians working outside hospital settings

The lower preference for in-person training among physicians may reflect differences in work organization, time flexibility, and access to training opportunities. In-person formats often require dedicated time and physical presence, which may be less feasible in certain clinical contexts, particularly in primary care or decentralized services. One possible explanation is that physicians working outside hospital settings may have fewer opportunities to attend scheduled in-person training during working hours. This may help explain why more flexible formats could be perceived as more suitable in these settings. Previous research has also shown that IPV training varies across professional groups and clinical settings, suggesting that training needs and opportunities may differ depending on professional and organizational context (21).

### Implications for training design

Overall, the findings indicate variation in training preferences across professional groups. These findings may be relevant when considering future training initiatives. Evidence from systematic reviews suggests that blended learning approaches, which combine face-to-face and digital components, may enhance learning outcomes and better accommodate diverse educational needs (24, 25). Combining in-person training formats with digital components that enhance accessibility may increase engagement across diverse clinical contexts. Such blended approaches are also reflected in IPV training literature, where multiple delivery formats are commonly integrated within interventions (26). However, the current evidence does not establish the comparative effectiveness of specific modalities. Importantly, the present study explored training preferences rather than training effectiveness. Therefore, no conclusions can be drawn regarding whether preferred training formats are more effective in improving knowledge, self-efficacy, reporting behavior, or patient outcomes. This distinction is important, as previous evidence from related areas of violence care suggests that targeted education can improve healthcare professionals’ knowledge and confidence (13). Future research should examine how different training formats influence not only knowledge outcomes, but also self-efficacy, clinical practice, reporting behavior, and patient outcomes over time. Longitudinal and intervention-based studies are particularly needed to determine whether specific training approaches lead to sustained improvements in mandatory reporting of IPV.

## Limitations and strengths

Several limitations should be considered. First, the study is based on a non-probability sampling strategy, which may limit the generalizability of the findings. The survey was distributed through selected hospital trusts, higher education institutions, professional networks, and social media, and we do not have information on how many individuals were exposed to the invitation. As a result, a response rate could not be calculated, and the risk of selection bias cannot be assessed. Participation was voluntary, introducing a risk of self-selection bias, as individuals with a particular interest in IPV or training may have been more likely to participate. And the sample consisted predominantly of women and nurses, which may limit the transferability of the findings to other professional groups within healthcare. Differences in training preferences across professions should therefore be interpreted with caution.

Second, the questionnaire was intentionally kept brief to facilitate participation among busy healthcare professionals. While this likely contributed to complete responses (i.e., no missing data), the brevity of the instrument may have limited the depth and nuance with which key constructs were assessed. In particular, training preferences and self-efficacy—including both perceived skills for participating in training and perceived skills in mandatory reporting of IPV—were captured using relatively simple measures. This may have reduced the ability to fully reflect the complexity of these constructs.

Third, the cross-sectional study design precludes causal inferences about relationships between participant characteristics and training preferences, and the findings should not be interpreted as evidence of the effectiveness of specific training formats. Reported preferences do not necessarily translate into improved knowledge, self-efficacy, reporting behavior, or outcomes for individuals exposed to IPV. Future studies are needed to determine whether the training formats preferred by participants are also effective in improving professional practice and patient outcomes.

Finally, due to limited prior evidence to guide variable selection, multiple variables were included in the regression analyses without formal adjustment for multiple testing. This may have increased the risk of Type I errors, while the relatively modest sample size may have increased the risk of Type II errors. The analyses should therefore be considered exploratory, and findings interpreted accordingly.

The study was conducted in Norway, and the findings may not be directly transferable to healthcare systems with different legal frameworks and organizational structures.

## Conclusion

This study explored healthcare professionals’ preferences for training formats on mandatory reporting of intimate partner violence. In-person meetings, courses, and workshops were the most frequently preferred formats. Digital formats such as e-learning and webinars were also widely endorsed, particularly among professionals reporting time constraints. Less experienced professionals appeared more likely to prefer video-based and online formats, while physicians and professionals working outside hospital settings were less likely to prefer in-person training. These findings highlight variation in training preferences across healthcare professionals. Such variation may be relevant when considering the delivery of future training initiatives on mandatory reporting of IPV.

## Data Availability

The raw data supporting the conclusions of this article will be made available by the authors, without undue reservation.
